# From Solutions to Photovoltaic Devices: Assessing the Impact of Zn Complexation for Bifacial Cu_2_ZnSn(S,Se)_4_ Thin Films

**DOI:** 10.1021/acsaem.5c02266

**Published:** 2025-10-08

**Authors:** Alice Sheppard, Jacques Kenyon, Nada Benhaddou, Lila Mahmoud, Alexander W. Black, Jake W. Bowers, David J. Fermin

**Affiliations:** † School of Chemistry, 1980 University of Bristol, Cantocks Close, BS8 1TS Bristol, U.K.; ‡ H. H. Wills Physics Laboratory, University of Bristol, Tyndall Avenue, BS8 1TL Bristol, U.K.; § Centre for Renewable Energy Systems Technology (CREST), Wolfson School of Mechanical Electrical and Manufacturing Engineering, 5156 Loughborough University, Loughborough LE11 3TU, U.K.

**Keywords:** photovoltaic devices, thin-film devices, selenium incorporation, dimethylformamide

## Abstract

Solution-based deposition of high-quality inorganic compound semiconductors onto a variety of substrates is a key challenge toward integrating photovoltaic technologies into a wide range of infrastructures. Cu_2_ZnSn­(S,Se)_4_ (CZTSSe) is one of the most promising solar absorbers processable by solution-based methods, however there is a substantial knowledge gap linking the chemistry of cation complexes and chalcogen precursors, e.g. thiourea (TU), to the microstructure and opto-electronic properties of the thin-films. In this study, we focus our attention on the complexation of zinc chloride (ZnCl_2_) and zinc acetate (ZnAc_2_) in dimethylformamide (DMF)-based CZTS precursor inks and how this ultimately affects the performance of CZTSSe thin-film devices on F/SnO_2_ (FTO) substrates. Acetate coordination not only improves the overall CZTSSe composition and structural uniformity but also lowers the zinc salt decomposition temperature, from 519 to 284 °C, which significantly affects the rate of grain growth during selenization and final microstructure. ZnAc_2_-based CZTSSe films show densely packed grain growth due to the fast rate of selenium incorporation during annealing, while ZnCl_2_-based CZTSSe displays slower rates due to the high decomposition temperature of ZnCl_2_. Upon the incorporation of 25 nm Mo at the CZTSSe/FTO interface, a champion power conversion efficiency of 6.02% was achieved with ZnAc_2_ precursor salt, over two times greater than the equivalent device architecture prepared with ZnCl_2_, at 2.62%. This investigation illustrates the significant role of the molecular complexes in tuning the grain growth kinetics and final microstructure and therefore improving device performance on semitransparent substrates.

## Graphical abstract

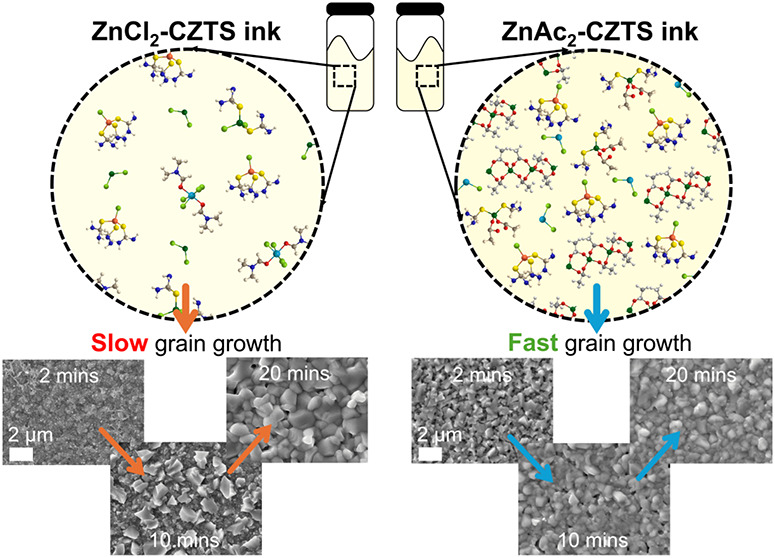

## Introduction

The decarbonization of electricity generation is pivotal to meet legally binding commitments to net carbon zero by 2050.^1^ A key area of industrial growth is the implementation of building-integrated photovoltaic (BIPV) modules, typically semitransparent, flexible, and bifacial applications. A promising thin-film PV material for such technologies is Cu_2_ZnSn­(S,Se)_4_ (CZTSSe) due to its earth-abundant base elements, low cost, and ability to be deposited on a wide range of substrates.^2–8^

Since 2024, several studies have reported solution-processed CZTSSe devices with certified power conversion efficiencies (PCEs) above 14%, reigniting efforts toward developing efficient, stable, scalable, and cost-effective PV technology.^9–14^ Solution-processing commonly involves precursor inks based on Cu, Sn and Zn metal salts in solvents such as dimethylformamide (DMF) and dimethyl sulfoxide (DMSO), in the presence of thiourea (TU) acting sulfur precursor and complexing agent.^15^ Significant improvements to CZTSSe efficiencies have been brought about by tuning the properties in the molecular precursor ink, including the choice of solvent, Cu–Sn oxidation states, cation alloying, or extrinsic doping.^11,16–24^ The solution processing of the thin film involves sequential spin coating and drying steps of inks onto Mo-coated glass, generating a dry Cu_2_ZnSnS_4_ (CZTS) precursor film, which is subsequently selenized via reactive annealing. This raises a key fundamental question, *how solvation and complexation chemistry in the inks can have an impact on the elemental ordering of polycrystalline CZTSSe annealed at temperatures in the range of* 550 °C? We have recently shown that molecular complexes in the ink assemble into submicron-size aggregates in solution, which affect the microstructure of dry precursor, the morphology of the polycrystalline thin-film, and its PV performance.^25^ We have also demonstrated how blending various proportions of DMF and isopropanol (IPA) directly affected Sn distribution, local work function landscape, and device performance of F/SnO_2_ (FTO)-based CZTSSe.^26^

In addition to the nature of the solvent, the chemistry of the metal salts has also been linked to significant changes in the PV performance. Common counterions for the metal precursor include chloride (Cl^–^), acetate (Ac^–^), and nitrate.^27–31^ TU stabilizes Cu^+^ in solution, forming Cu­(TU)_1–4_Cl through S,^32–35^ while ZnCl_2_(TU)_2_, ZnAc_2_(TU)_2_, and SnCl_2_(TU) complexes have also been reported in the literature.^36,37^ Instead of complexing with TU, SnCl_4_ coordinates with DMF or DMSO, forming SnCl_4_(DMF)_2_ and SnCl_4_(DMSO)_2_, respectively.^16,30^ Trifiletti et al.^38^ proposed that metal acetates form an intermetallic network between cation centers linked by TU in DMSO, generating a sol–gel with homogeneous distribution of metals upon deposition. Ahmad et al.^39^ compared metal chloride and acetate precursors in the synthesis of Cu_2_ZnSnS_4_ nanoparticles (NPs) at 250 °C. This study concluded that there was negligible incorporation of Zn into the NPs for chloride-based metal precursors due to the strong adsorption of Cl^–^ ions onto ZnS NPs, whereas acetate-based metal salts lead to the formation of Cu_2_ZnSnS_4_ NPs. A recent work by Moser et al.^29^ concluded that Sn loss during the reactive annealing was mitigated by substituting zinc chloride (ZnCl_2_) for zinc acetate (ZnAc_2_). These observations appear consistent with the fact that high-efficiency CZTSSe devices often use CuCl, SnCl_4_, and ZnAc_2_ as precursors.^9–14^ However, a link between the coordination chemistry of the precursor salts in solution and the PV performance is fundamentally missing.

In this work, we provide the first in-depth analysis of the link between complexation in molecular precursors featuring ZnCl_2_ and ZnAc_2_, their thermochemistry, and their thin-film microstructure, composition, and PV device performance. Considering the applications of CZTSSe in BIPV, FTO substrates have been utilized in this work. The chemical nature of the Zn salt has clear effects on cation complexation and thermochemical properties of the precursor inks, which are linked to stark differences in grain growth kinetics and CZTSSe film microstructure. Interestingly, these differences have little effect on the surface composition and electronic landscape as probed by XPS and characterized by energy-filtered photoemission electron microscopy (EF-PEEM). These observations suggest that the nature of the Zn counterions has an influence on the bulk properties of the materials. We also observed that the deposition of a 25 nm Mo layer at the FTO surface leads to a significant improvement in device PCE from 2.62% to 6.02%, which further emphasizes the complexity of nucleating high-quality CZTSSe.

## Results and Discussion

### Complexation with Chloride and Acetate Zinc­(II) Precursor Counterions

The precursor solutions are prepared with CuCl_2_, SnCl_2_, either ZnCl_2_ or ZnAc_2_, and TU salts dissolved in a 75:25 ratio of dimethylformamide/isopropanol (DMF/IPA) (Supporting Information—Experimental Methods) and a TU/(Cu+Zn+Sn) of 5, as developed from our previous study for CZTSSe on an FTO substrate.^26^ Figure 1 displays the Fourier transform infrared (FTIR) spectra of individual metal salts with TU (TU+MX_2_, where M is the metal ion and X is the precursor counterion −Cl or Ac) as well as the complete molecular precursor solutions (ZnX_2_-pre). The various IR modes of DMF, TU, and IPA are also summarized in Table S1.^40,41^ Figure 1a shows FTIR spectra over the fingerprint region (600–1800 cm^–1^), indicating that the main changes in complexation occur through the TU C=S bond at 740 cm^–1^. With the addition of CuCl_2_·2H_2_O, the appearance of the peak at 720 cm^–1^ and decreased intensity of free TU at 740 cm^–1^ indicate the formation of Cu-TU and Lewis acid–base interaction.^42^ This bathochromic shift is due to a decrease in the C=S bond order. Furthermore, no significant changes in the TU H–N–H bending mode at 1615 cm^–1^ are observed, confirming that TU–metal complexation takes place via sulfur bonds.^43^ In Figure 1b, no change in free TU peak is detected with the addition of SnCl_2_, showing evidence that SnCl_2_ is not complexed by TU. Assessing TU+ZnX_2_ in Figure 1b shows a small change in the relative intensity between free TU C=S at 740 cm^–1^ and the emergence of a peak at 720 cm^–1^. Compared with TU+CuCl_2_, there is only a slight decrease in TU intensity, indicating that most TU is uncoordinated in solution. The shoulder peak at 720 cm^–1^ may be associated with ZnCl_2_(TU)_2_ or ZnAc_2_(TU)_2_ complexes, as reported in the literature.^36,37,44–46^ This may allow additional coordination environments to be present, as discussed below.

**Figure 1:**
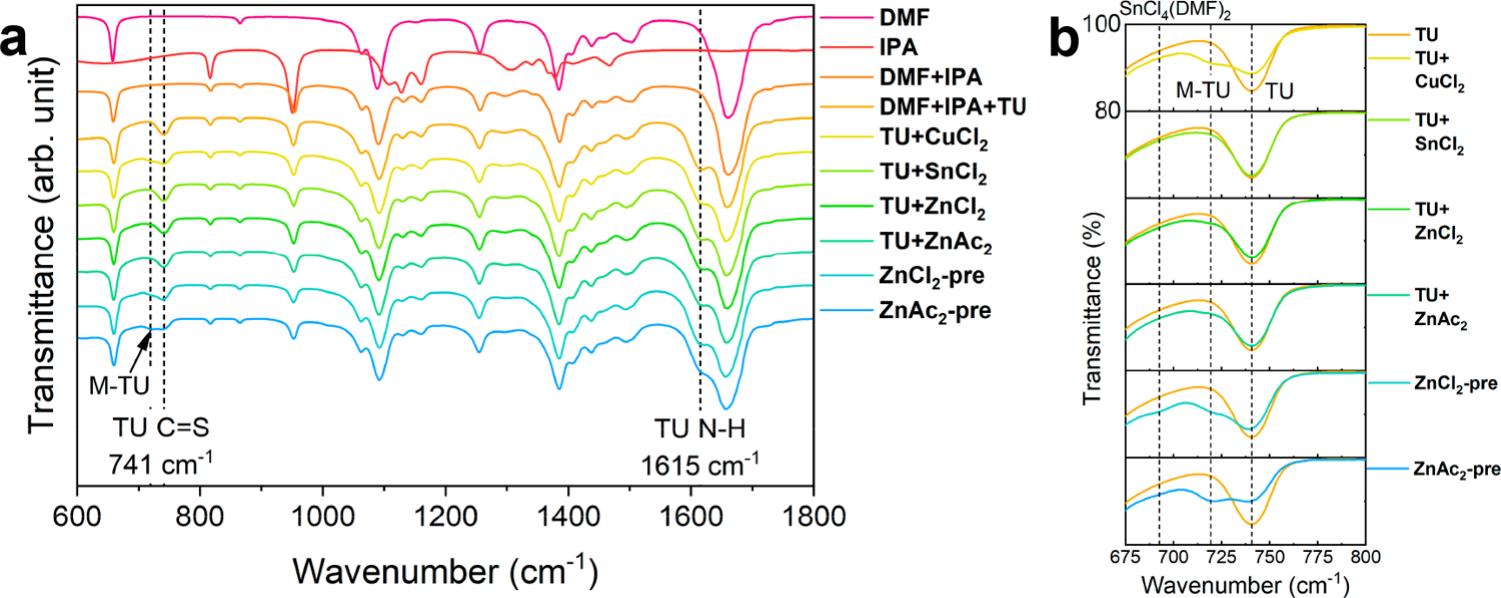
FTIR spectroscopy analysis, between 600 and 1800 cm^–1^, of the individual metal salts with TU (TU+MX_2_) and the complete molecular precursor solutions prepared with ZnCl_2_ (ZnCl_2_-pre) and ZnAc_2_ (ZnAc_2_-pre) precursor salts (a). TU C=S stretching in DMF/IPA solvent mixture in presence of each individual metal salts (TU +MX_2_) and the complete precursor solutions (ZnX_2_-pre) (b). TU, M–TU, and SnCl_4_(DMF)_2_ peaks are labeled at 740, 720, and 693 cm^–1^, respectively.

Yang et al.^47^ characterized the coordination of ZnAc_2_ in water based on the wavenumber spacing between the symmetric (*ν*_s_) and asymmetric stretching (*ν*_a_) of the carboxyl-group in FTIR spectra (Δ*ν*_a–s_). Δ*ν*_a–s_ can be related to various coordination environments, one of which is bidentate bridging coordination, which describes where the two acetate oxygen atoms bond to two different metal centers. According to Trifiletti et al.,^38^ bidentate bridging coordination of acetate ions is preferred and is solvent-independent. Due to this, we propose that acetate ions will have the same bridging coordination environment in DMF as described in the literature. This additional Zn coordination environment can directly impact the speciation with free TU, CuCl­(TU)_3_, and Sn in the sol–gel. An additional peak at 692 cm^–1^ can be observed for ZnCl_2_-pre, which correlates to the SnCl_4_(DMF)_2_ complex. The existence of this SnCl_4_(DMF)_2_ complex in ZnCl_2_-pre and not in ZnAc_2_-pre implies that Sn is more freely able to coordinate with DMF without bridged acetate counterions present in the solution. This suggests that ZnAc_2_ bidentate bridging coordination facilitates an interaction between Sn metal centers, CuCl­(TU)_3_, and ZnAc_2_(TU)_2_. This is an important observation and has an impact on the compositional inhomogeneity in the CZTS precursor films, as discussed further below.

Based on our findings, as well as those reported in the literature, we propose the complexes formed in the ZnCl_2_- and ZnAc_2_-based precursor solutions, which are displayed in Figure 2. While CuCl­(TU)_3_, ZnCl_2_(TU)_2_, and ZnAc_2_(TU)_2_ are tetrahedrally coordinated (Figure 2a–c),^15,19,44,48^ SnCl_4_(DMF)_2_ show an octahedral coordination (Figure 2e). Figure 2d shows the proposed structure of ZnAc_2_ complex featuring bidentate bridging coordination,^47^ which is in stark contrast to the ZnCl_2_ complex in which the Cl^–^ groups remain strongly bound.

**Figure 2:**
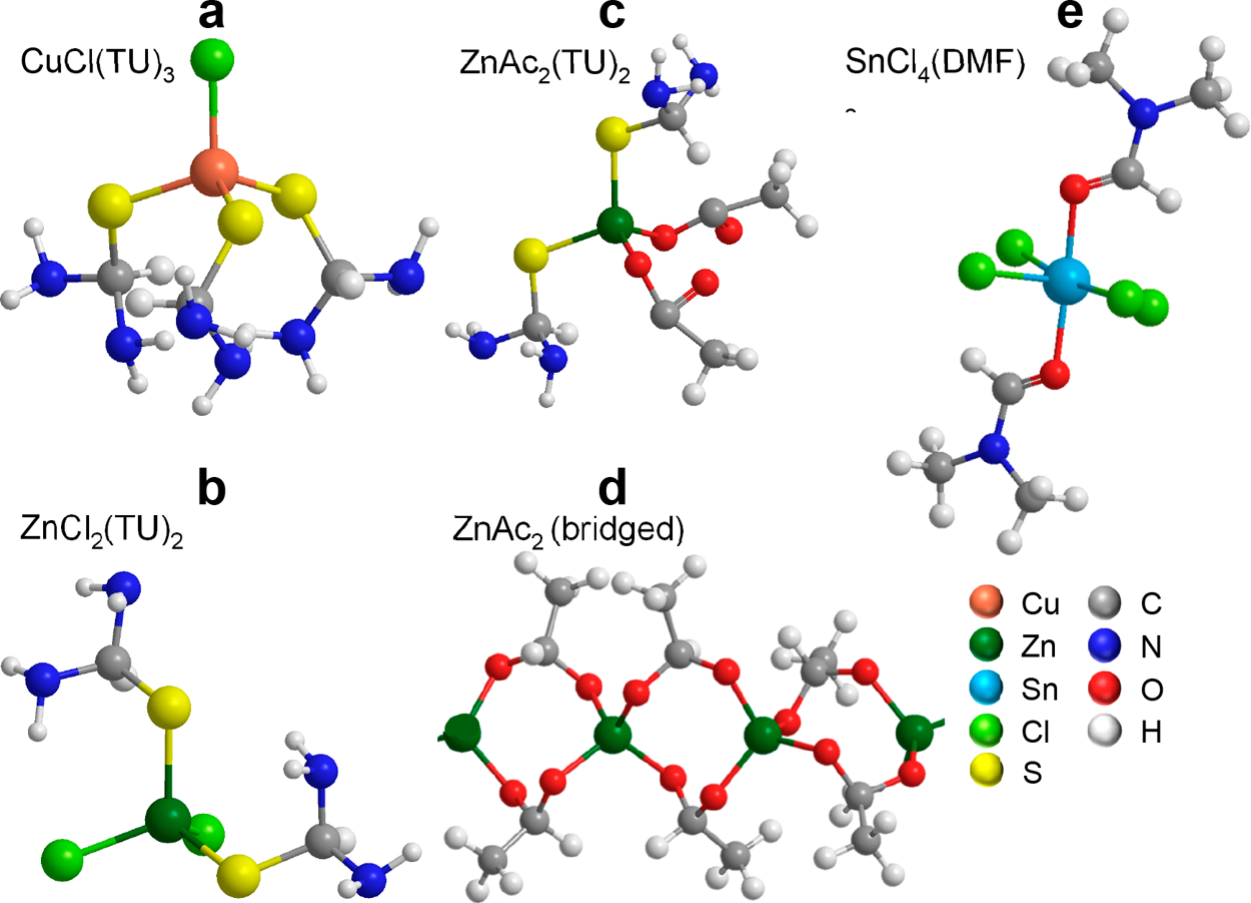
Proposed structure of CuCl­(TU)_3_ (a), ZnCl_2_(TU)_2_ (b), ZnAc_2_(TU)_2_ (c), ZnAc_2_-bridged coordination (d), and SnCl_4_(DMF)_2_ (e) complexes.^5,19,44,47,48^

Thermogravimetric analysis (TGA) responses of the dried ZnCl_2_- and ZnAc_2_-based precursor solutions (methodology described in Experimental Details) are shown in Figure 3, with the TGA of individual precursor salts displayed in Figure S1 and summarized in Table S2. Three general regimes can be identified in the thermal processing of CZTS precursors: (1) dehydration of complexes from room temperature to 180 °C; (2) decomposition of TU-M complexes and metal sulfide formation between 200 and 270 °C, and (3) metal oxide formation from 275 °C.^36^ According to the trend in Figure 3, DMF, TU, and TU-M complexes decompose from room temperature to 250 °C (see also Figure S1e). From 250 to 550 °C, chloride and acetate impurities are removed.^22,36^ For the dried precursor salts, there are additional mass losses that cannot be associated solely with the removal of organic complexes and impurities, at ∼285 and ∼510 °C for ZnAc_2_-pre and ZnCl_2_-pre, respectively. When assessing the decomposition of the individual salts (Figure S1 and Table S2), ZnAc_2_ decomposes at a lower temperature (284 °C) than ZnCl_2_ (519 °C). ZnCl_2_ and ZnAc_2_ decompose into ZnO,^49–51^ which accounts for the 7% and 6% residue in Figures S1c,d. The additional mass losses in the dried precursor solutions can therefore be related to the decomposition of ZnAc_2_ and ZnCl_2_. These results are consistent with FTIR spectroscopy, as most of the ZnCl_2_ and ZnAc_2_ salts do not complex with TU (Figure 1b). During spin coating, a hot plate temperature of 350 °C was used, which is lower than the decomposition temperature of ZnCl_2_ but higher than of ZnAc_2_. This key difference is likely to affect the CZTSSe film formation during reactive annealing. No additional decomposition peaks can be directly associated with CuCl_2_·2H_2_O and SnCl_2_ salts in either ZnCl_2_-pre or ZnAc_2_-pre (Figures S1a,b). The mass loss at 600–650 °C can be associated with the removal of SnS and S_*x*_.^52^ Interestingly, SnS loss occurs at slightly higher temperatures in the case of ZnAc_2_-pre, which provides further evidence of the stronger interaction of Sn complexes with other cations in this formulation.

**Figure 3:**
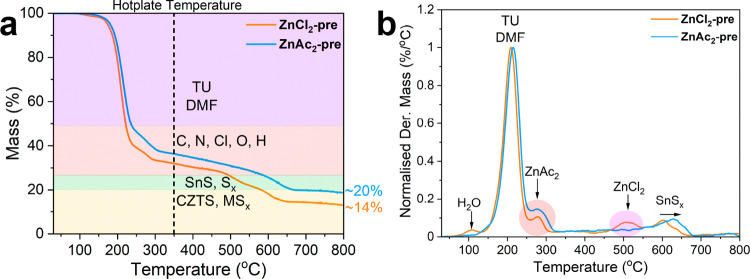
TGA profiles of the dry precursors ZnCl_2_-pre and ZnAc_2_-pre (a). DMF, H_2_O, and TU decomposition is shaded in purple, chloride and acetate decomposition is shaded in red, SnS and S_*x*_ are shaded in green, and CZTS and metal sulfide formation is shaded in yellow. Normalized derived mass (Der. Mass) of each precursor solution with respect to temperature (b), with the decomposition of ZnAc_2_ (red) and ZnCl_2_ (pink) highlighted.

### Precursor Counterion Dependence on Grain Growth Kinetics

Figure 4 shows a collection of top-down and cross-sectional scanning electron microscope (SEM) images obtained at various annealing times at 530 °C of ZnCl_2_- and ZnAc_2_-based CZTSSe absorbers. The direct formation from amorphous CZTS to crystalline CZTSSe is demonstrated for both precursor counterions, without evidence of secondary phases, such as Cu_2–*x*_(S,Se), Cu_2_Sn­(S,Se)_3_ (CTS), and Zn­(S,Se).^23,53^ In the case of ZnCl_2_–CZTSSe, grain growth begins after 5 min of selenization (Figure 4a), where the grain size gradually increases with increasing annealing time. For ZnAc_2_–CZTSSe, grain growth begins immediately after reaching 530 °C (0 min) (Figure 4c). Unlike ZnCl_2_–CZTSSe, grains increase in size up to 5 min, with a low grain packing density, before decreasing from 10 to 30 min of annealing. After 20 min of selenization, films prepared with either Zn counterion reach a compact structure, minimizing the risk of shunting during device fabrication, with ZnCl_2_–CZTSSe having larger grains than ZnAc_2_–CZTSSe.

**Figure 4:**
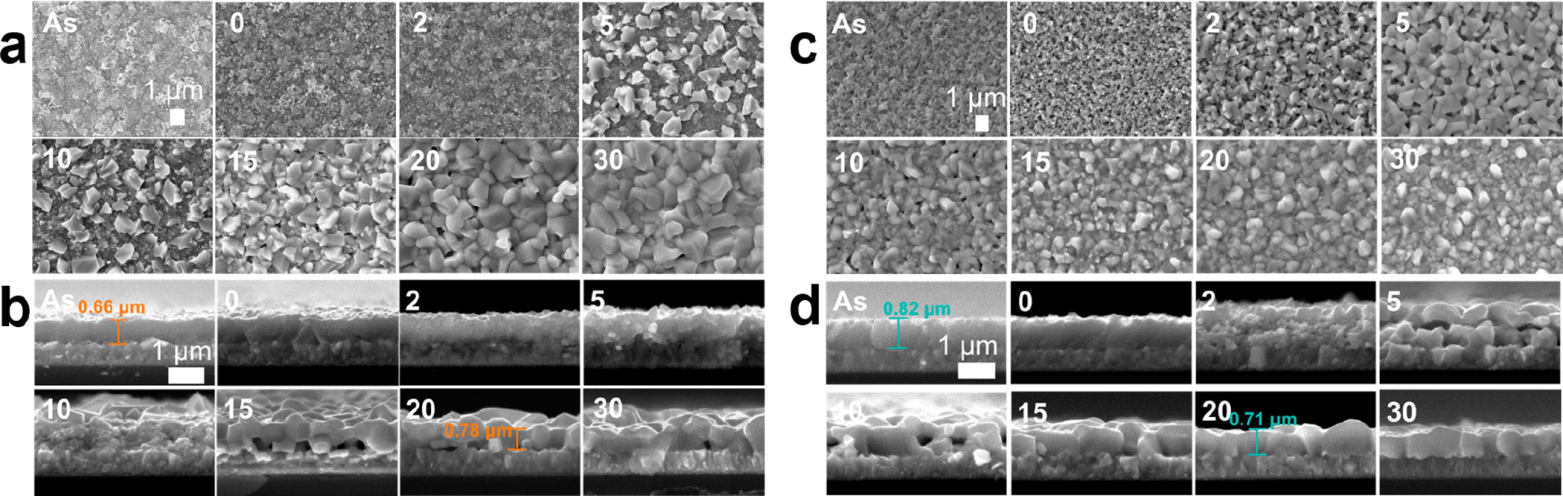
Top-down (a, c) and cross-sectional (b, d) SEM of ZnCl_2_- (a, b) and ZnAc_2_-based (c, d) CZTSSe absorbers on FTO substrates during selenization. The number in each panel represents the annealing time in minutes at 530 °C. The scale bar for each condition is the same as that for the as-deposited (As) image.

Cross-sectional SEM images show that the as-deposited ZnAc_2_ precursor film is thicker than the ZnCl_2_ precursor film, at 0.82 and 0.66 μm, respectively, despite using the same spin coating conditions and solution concentration (Supporting Information—Experimental Methods). As shown in Figure 3a, the residual mass of ZnAc_2_-pre is greater than that of ZnCl_2_-pre, at 20% and 14%, respectively, indicating that using a ZnAc_2_ salt leads to a more thermally stable precursor solution. This higher residual mass may be contributing to the greater overall thickness of the ZnAc_2_ precursor film after spin coating and drying at 350 °C. Top-down grain growth occurs in both cases, as shown in Figure 4b,d, initially forming sharp-faceted grains at the top and a fine-grain bottom layer, leading to top-to-bottom grains. Figure S2 illustrates the evolution of the surface morphology at various annealing times, as probed by high-resolution confocal laser scanning microscopy (CLSM). These images identify quantifiable changes in surface roughness at the nanoscale, in a field of view of 130 μm × 130 μm. We can see that the root-mean-square height (S_q_) of the films is below 100 nm over the field of view, demonstrating the homogeneous nature of the film deposition. Figure 5a contrasts the time evolution of S_q_, showing an increase from 64 to 116 nm in the case of ZnCl_2_–CZTSSe through the course of selenization. On the other hand, the ZnAc_2_–CZTSSe precursor shows a sharp increase in S_q_ from 46 to 110 nm after 5 min of annealing, followed by a decrease to 52 nm after 30 min of annealing. Based on the evolution of the surface roughness (Figure 5a) and grain microstructure (Figure 4), we propose the grain growth mechanism illustrated in Figure 5b. The observations suggest that the same ZnCl_2_–CZTSSe film morphology obtained after 10 min annealing is achieved only under 2 min in the case of ZnAc_2_–CZTSSe. However, we must stress the fact that this analysis is qualitative, given the complex configuration of the reactive annealing step.

**Figure 5:**
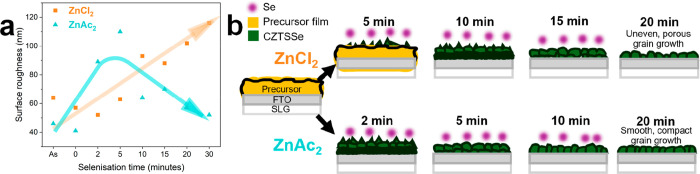
Surface roughness (a) and grain growth mechanism (b) of ZnCl_2_- and ZnAc_2_-based CZTSSe absorbers on FTO substrates during selenization.

We propose that the faster selenization rate of ZnAc_2_–CZTSSe is linked to the difference in complexation and extent of decomposition of the Zn precursor salts achieved during drying between spin coating steps (Figures 1, 3, and S1c,d). As described in the Experimental Methods (Supporting Information), drying steps were carried out at a hot plate temperature of 350 °C. This temperature is significantly higher than the decomposition temperature of ZnAc_2_ (284 °C), enabling Zn^2+^ to associate with other cations and sulfur to form a disordered CZTS layer, which is swiftly transformed into high-quality CZTSSe upon annealing at 530 °C. On the other hand, the decomposition temperature of ZnCl_2_ is 519 °C, which is considerably higher than the drying temperature, hindering the formation of CZTS in the precursor layer and slowing down the selenization process and grain growth.

Figure 6 shows the X-ray diffraction (XRD) patterns and Raman spectra for the ZnCl_2_- and ZnAc_2_–CZTSSe absorbers during selenization. Polycrystalline CZTSSe with (112), (204)/(220) and (312)/(116) crystallographic planes at approximately 2θ = 27.3°, 45.3°, and 53.7° (JCPDS 04–019–1847), respectively, can be observed for both absorber conditions after 20 min of selenization. The enlarged image of the (112) diffraction peak (Figure 6c,d) shows a shift to lower diffraction angles with increasing selenization time. This shift is more gradual for ZnCl_2_–CZTSSe compared to ZnAc_2_–CZTSSe. This trend is also observed in the (112) diffraction peak full-width at half-maximum (fwhm) (Table S3). For ZnCl_2_–CZTSSe, the fwhm gradually decreases from 0.64° to 0.14° from 2 to 20 min, respectively. The fwhm of ZnAc_2_–CZTSSe sharpens after 5 min of selenization, at 0.16°, which further supports the notion of increased selenization rate when using an acetate-based Zn counterion. Both absorber conditions display a peak at 2θ = 14.5°, which can be associated with SnSe_2_ and is commonly detected for CZTSSe on FTO substrates.^54–56^ Raman spectra shown in Figure 6e,f indicates that the reaction pathway is independent of the zinc precursor counterion, directly transforming from amorphous CZTS to highly crystalline CZTSSe, rather than through binary or ternary phases, shown by the coexistence of low- (Se modes—173, 196, 233, and 245 cm^–1^) and high-frequency Raman peaks (S modes—328 cm^–1^).

**Figure 6:**
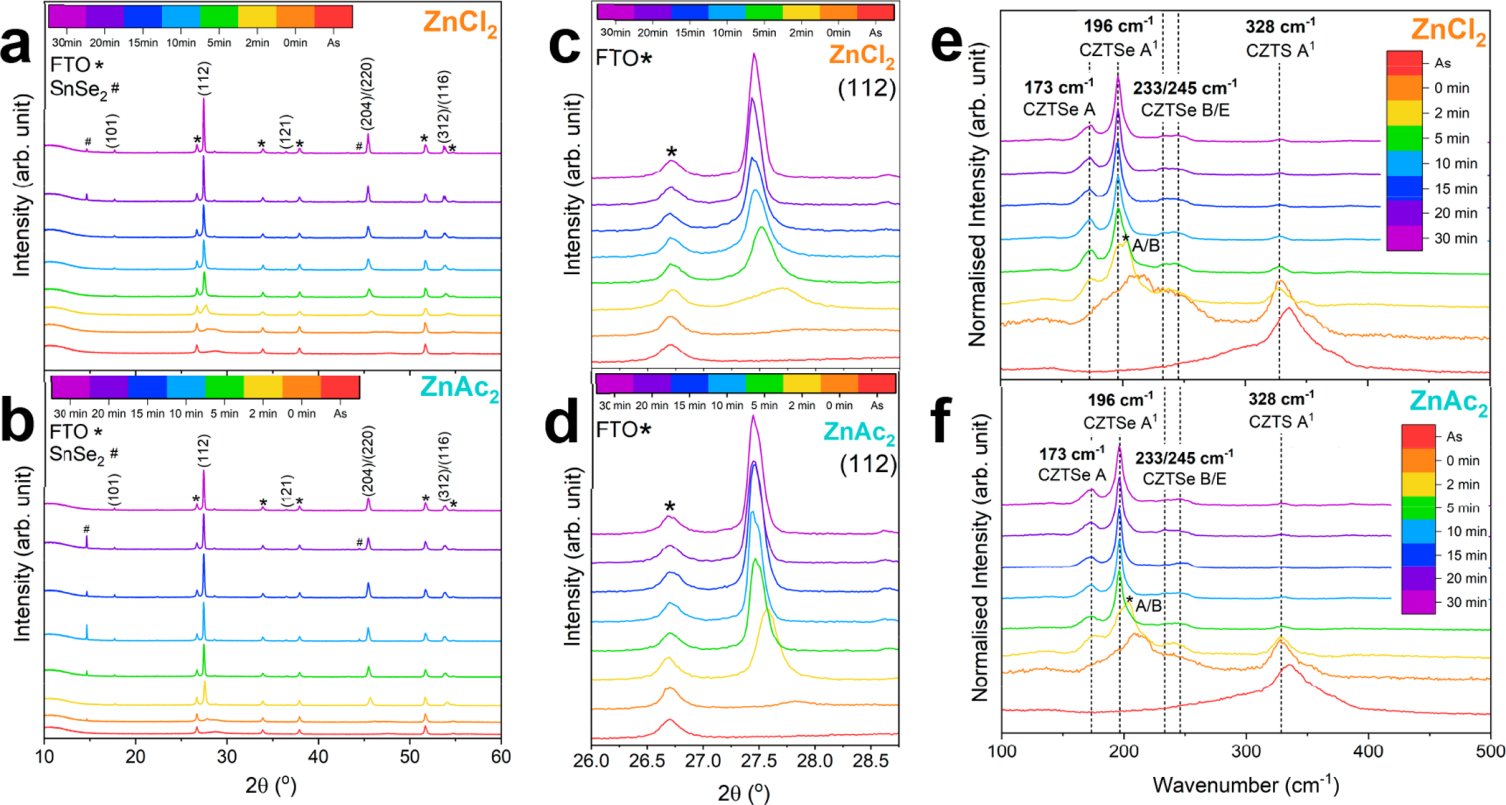
XRD patterns (a–d) and Raman spectra excited with 488 nm laser (e, f) of ZnCl_2_- (a, c, e) and ZnAc_2_-based (b, d, f) CZTSSe absorbers on FTO substrates during selenization: As (red), 0 min (orange), 2 min (yellow), 5 min (green), 10 min (light blue), 15 min (blue), 20 min (purple), and 30 min (magenta).

The orbital splitting and binding energy positions of Cu 2p, Zn 2p, and Sn 3d, measured with X-ray photoelectron spectroscopy (XPS), confirmed Cu^+^, Zn^2+^, and Sn^4+^.^57^ Using ZnAc_2_ leads to an increase in Zn/Sn surface composition compared to ZnCl_2_, at 1.53 and 1.09, respectively (Figure 7a–c and Table S4). Energy-filtered photoemission electron microscopy (EF-PEEM) analyzed the surface spatial variation of the work function (WF) of the CZTSSe absorber (Figure 7d–g). The maps show a relatively smooth WF distribution across the field of view for both precursor formulations. In previous studies, we have observed that surface Sn disorder can generate areas of low WF.^20,21,58^ The distribution of WF in the case of ZnCl_2_–CZTSSe appears slightly narrower than the ZnAc_2_–CZTSSe case, with the center shifted by approximately 30 meV toward lower values. The lower WF center could be linked to the lower Zn/Sn ratio, which agrees with our previous study where we observed the WF centre decreasing to values as low as 4.9 eV at Zn/Sn < 1 as a result of changes in DMF/IPA ratio.^26^

**Figure 7:**
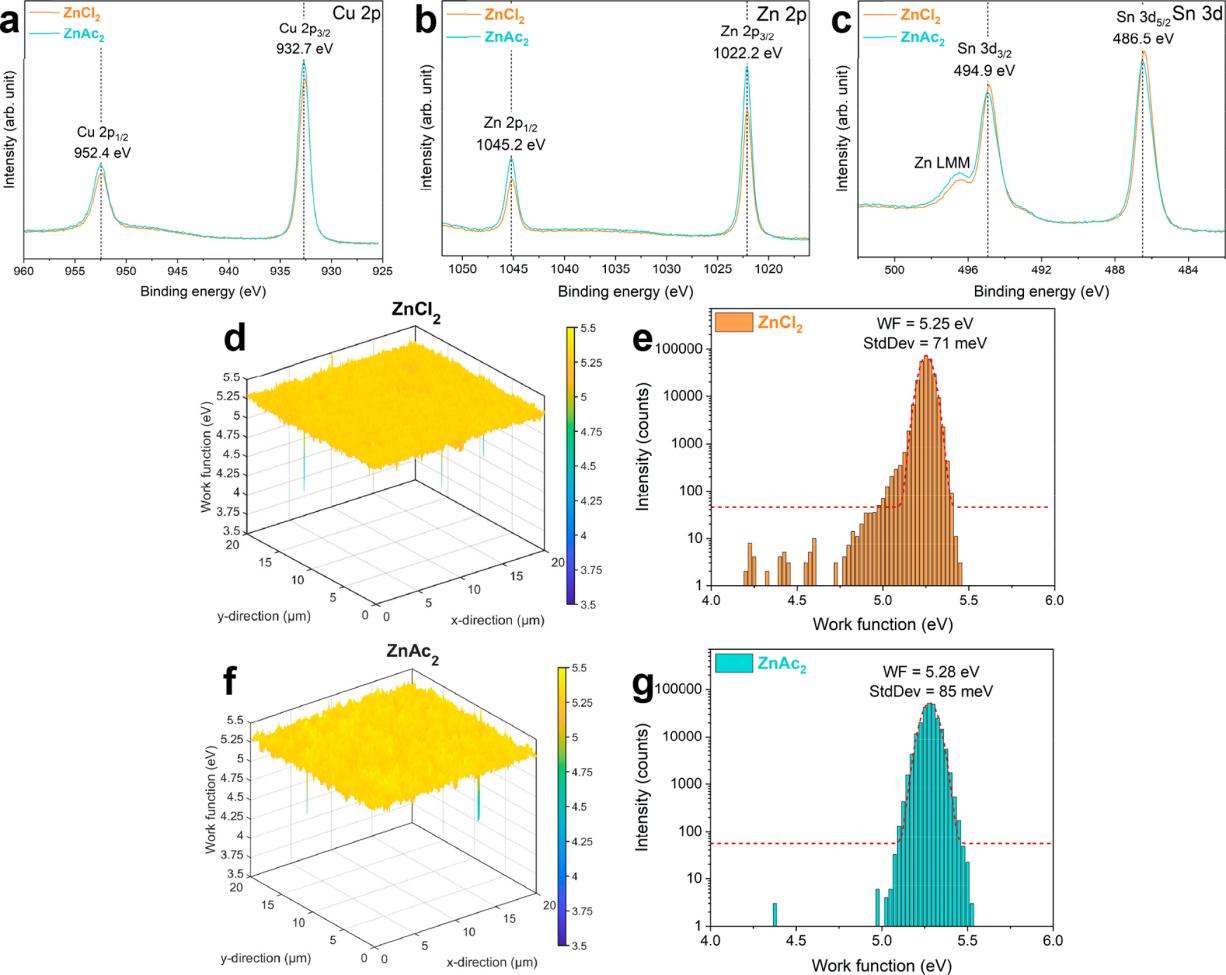
XPS spectra of Cu 2p (a), Zn 2p (b), and Sn 3d (c) and 3D work function (WF) maps (d, f) and corresponding distributions (e, g), measured by EF-PEEM, of ZnCl_2_- (orange) and ZnAc_2_–CZTSSe (aqua).

### The Effect of Zn Precursor and Substrate Composition in Device Performance

Following the procedure described in the Experimental Methods (Supporting Information), 0.25 cm^2^ devices were fabricated in the substrate configuration stack SLG/FTO (500 nm)/Mo (0 or 25 nm)/CZTSSe/CdS (50 nm)/i-ZnO (50 nm)/Al/ZnO (500 nm)/Ag (500 nm). In addition to assessing the effect of the Zn precursor, we also investigated the role of a 25 nm Mo layer sputtered on the FTO substrate. Figure S3 shows the morphology of the CZTSSe film and devices prepared with the ZnCl_2_ and ZnAc_2_ formulation, without (0) and with 25 nm Mo (25) at the back contact, labeled as 0-ZnCl_2_, 25-ZnCl_2_, 0-ZnAc_2_, and 25-ZnAc_2_, respectively. Interestingly, the grain size decreases in the presence of 25 nm Mo for both precursor counterions. Analyzing over a larger topographical area and with CLSM, there are inhomogeneities across the surface for ZnCl_2_-based absorbers which are not present for ZnAc_2_-based absorbers (Figure S3). Engberg et al.^59^ demonstrated the existence of surface patterning while using an all-chloride CZTS molecular ink. These inhomogeneities may be related to the Marangoni effect, which occurs due to variations in the surface tension. The lateral flow of the solution on the surface is dependent on ink properties, such as solute concentration, viscosity, and solvent evaporation rate. The reduction of such patterning for ZnAc_2_-based absorbers indicates that ZnAc_2_ stabilizes wettability gradients during drying, possibly due to the bidentate bridging coordination between acetate groups discussed earlier. Figure S3e–h shows the cross-sectional morphology of ZnCl_2_- and ZnAc_2_-based devices, indicating the CdS and transparent conducting oxide coatings uniformly cover the CZTSSe absorbers. With the inclusion of 25 nm Mo, a thin fine-grain layer forms at the back contact (Figure S3f,h), suggesting that Mo impacts the CZTSSe grain growth at the rear interface.

The *J*–*V* curves for the front-illuminated champion cells are shown in Figure 8a and summarized in Table S5, with the corresponding external quantum efficiency (EQE) spectra in Figure 8b. As these devices have nominally two window layers, “front illumination” refers to illumination through the CdS side of the stack. ZnAc_2_–CZTSSe devices, without and with Mo, show a clear improvement in front illumination PCE compared to ZnCl_2_–CZTSSe devices, with champion PCEs of 3.27% and 4.49% for 0-ZnCl_2_ and 0-ZnAc_2_ devices, respectively. This is further supported by the average cell performance (Figure 8c and Table S6). The combination of a 25 nm Mo insertion layer and ZnAc_2_ precursor salt (25-ZnAc_2_) boosted the PCE to a champion of 6.02%, with *J*_sc_ of 26.1 mA cm^–2^, *V*_OC_ of 0.403 V, and FF of 57.3%. 25-ZnAc_2_ shows improvements in all PV metrics (Figure 8c–f and Table S6), particularly *V*_OC_ and FF, resulting in an average PCE two times greater than that for 0-ZnAc_2_. The series resistance (*R*_s_) is approximately 3 Ω cm^2^ for all CZTSSe conditions (Figure S4a), likely originating from the FTO substrate. For 25-ZnAc_2_, the shunt resistance (*R*_sh_) improves considerably to an average value of 212 Ω cm^2^ (Figure S4b), showing a suppression of leakage current and improved carrier collection when combining both ZnAc_2_ and a 25 nm Mo insertion layer. The improvement of *R*_sh_ for 25-ZnAc_2_ greatly reduces the ideality factor from 2.15 for 0-ZnAc_2_ to 1.63, as shown in Table S5. In addition, Figure S4c compares the champion CZTSSe devices illuminated from the front and back. Both ZnAc_2_-based devices show improved diode behavior and performance under back illumination compared to ZnCl_2_-based devices, with PCEs of ∼0.6%. Despite the decrease in *J*_sc_ due to reduced light transmission, the inclusion of 25 nm Mo enhances the quality of the rear interface of ZnAc_2_–CZTSSe, shown by improved *V*_OC_ and FF (Table S7), further illustrating the interplay between the precursor composition and substrate properties. The overall enhancement of device performance metrics with the nanoscale Mo layer on FTO substrates is consistent with previous reports.^5,6,8^

**Figure 8:**
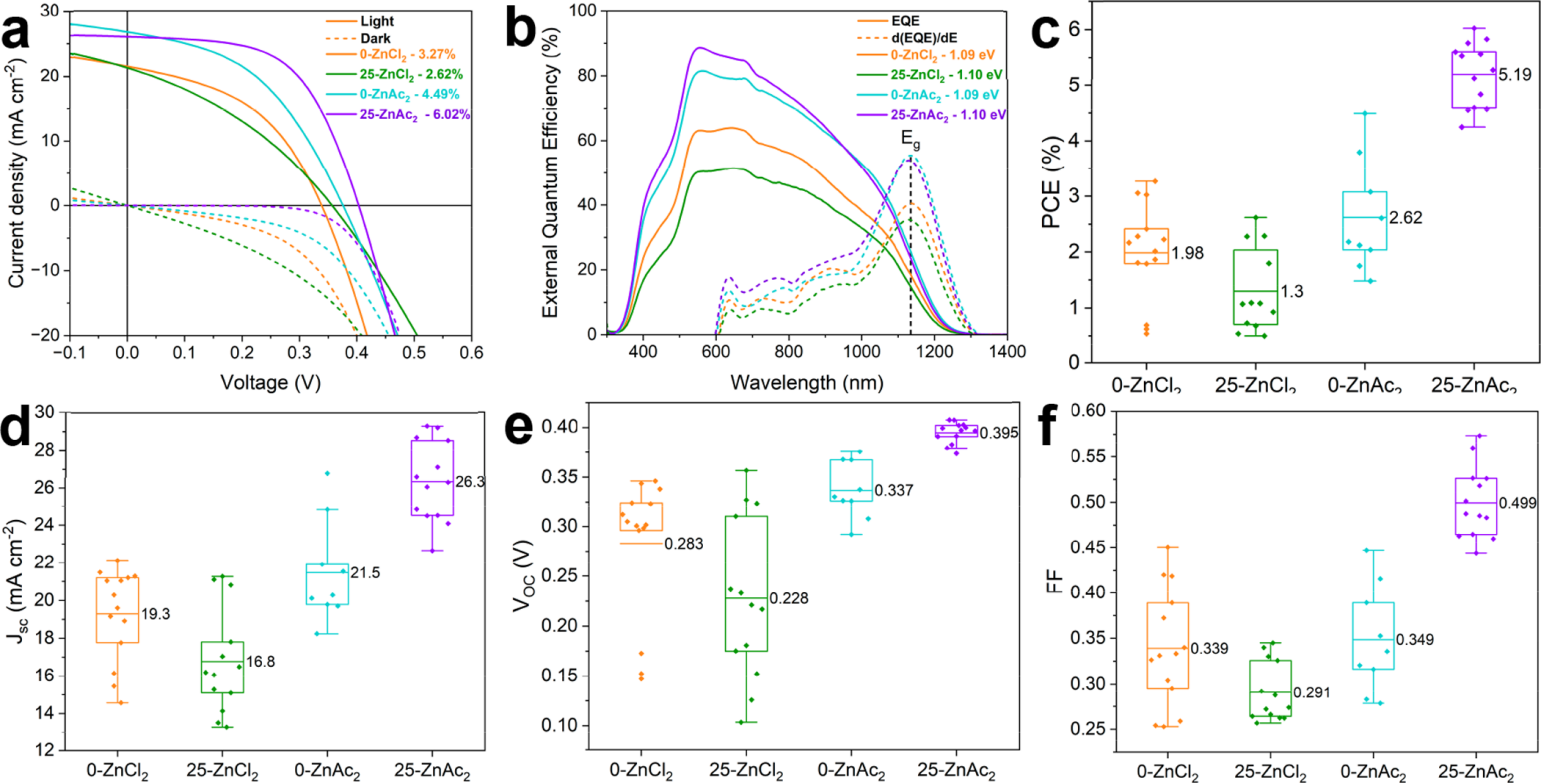
*J*–*V* and EQE measurements of champion cells (0.25 cm^2^) (a, b) for 0-ZnCl_2_ (orange), 25-ZnCl_2_ (green), 0-ZnAc_2_ (aqua), and 25-ZnAc_2_ (purple) CZTSSe devices. Box plots of PCE (c), *J*_sc_ (d), *V*_OC_ (e), and FF (f).

As shown in Figure S3b, the 25-ZnCl_2_ absorber has severe Marangoni patterning and porosity, shown by the high surface roughness of 209 nm, over 2.5 times greater than 25-ZnAc_2_ (Figure S3). This high roughness can be associated with localized areas of porous grain growth, leading to shunting pathways to the FTO back contact and reducing *R*_sh_ to 27.6 ± 9.7 Ω cm^2^, despite having 25 nm Mo to act as a hole-selective transport layer and improve carrier collection. The overall uniformity is also poor for 0-ZnCl_2_, to a lesser extent (Figure S3a), indicating that the combination of this layer and the ZnCl_2_-based precursor solution significantly impedes the absorber microstructure and overall uniformity and therefore the PCE. This demonstrates the importance of tuning the precursor solution through the Zn­(II) precursor counterion to improve overall uniformity, CZTSSe microstructure, and grain growth kinetics.

Finally, the EQE response is shown in Figure 8b. The higher EQE response between 400 and 500 nm for ZnAc_2_-based CZTSSe devices indicates an improvement in the CZTSSe/CdS interface. This may be associated with the reduced S_q_ of ZnAc_2_-based absorbers compared to ZnCl_2_-based CZTSSe (Figure S3) that improves CdS growth. Furthermore, carrier collection is improved through the CZTSSe bulk prepared with a ZnAc_2_ salt. The bandgap for all devices is approximately 1.1 eV, which is consistent with a S/(S+Se) ratio of 0.2.^60^

## Conclusions

In this work, we have uncovered the impact of the nature of Zn salt precursor on the solution processing of CZTSSe thin films, from chemical speciation in solution, to grain growth kinetics, film morphology, and photovoltaic performance. Our infrared studies show that most TU does not coordinate with ZnCl_2_ and ZnAc_2_, and their decomposition can be followed by thermogravimetric analysis. The considerably lower decomposition temperature of ZnAc_2_ compared to ZnCl_2_, at 284 and 519 °C, respectively, directly increases the rate of grain growth during selenization, significantly improving the grain microstructure of ZnAc_2_–CZTSSe compared to ZnCl_2_–CZTSSe. Surface patterning in the thin-film morphology, originating from inhomogeneous surface tension (Marangoni effect), is more significant in the ZnCl_2_ than the ZnAc_2_ formulation. We linked this macroscopic observation to the bidentate bridging coordination of acetate counterions in solution, which led to improvement in the uniformity of the film composition and microstructure.

Finally, coupling ZnAc_2_–CZTSSe with a 25 nm Mo insertion layer led to a champion PCE of 6.02% on an FTO substrate, with improvement in all device metrics with respect to the ZnCl_2_–CZTSSe-based devices. Our analysis clearly shows how the composition of the molecular precursor can have a substantial impact across different length scales, from complexation and interactions with other precursors, to the surface wettability, thermolysis, and crystal growth. These observations are key, not only in the formulation of precursors for thin-film PV, but also in the broader context of solution processing of high specification compound semiconductors.

## Supplementary Material



## Data Availability

Data are available at the University of Bristol data repository, data.bris, at https://doi.org/10.5523/bris.1qflk9pgu1wcz2q62iytqxxjl6.

## Notes

Experimental Details; summary of FTIR modes of DMF, TU and IPA; TGA measurements of precursor salts; surface roughness analysis of CZTSSe absorbers prepared with ZnCl_2_- and ZnAc_2_-based precursor solutions; summary of CZTSSe (112) diffraction peak during selenization; summary of surface composition, WF and WF distribution; SEM and surface roughness images of CZTSSe absorbers without and with a 25 nm Mo layer; summary of photovoltaic parameters for champion devices; summary of mean photovoltaic parameters over a range of devices; and series and shunt resistances of solar cells (PDF)
