# Supplementary material for: From Solutions to Photovoltaic Devices: Assessing the Impact of Zn Complexation for Bifacial Cu2ZnSn(S,Se)4 Thin Films
Source: ACS Appl Energy Mater. 2025 Oct 8;8(20):15258–67. doi: 10.1021/acsaem.5c02266 (PMC12569970; doi:10.1021/acsaem.5c02266)
Supplement: Supplementary file 1 [file ae5c02266_si_001.pdf]

# Supporting Information

## From solutions to photovoltaic devices: assessing the impact of Zn complexation for bifacial $Cu_2ZnSn(S,Se)_4$ thin-films

Alice Sheppard[1,2], Jacques Kenyon[3], Nada Benhaddou[3], Lila Mahmoud[1], Alexander W. Black[1], Jake W. Bowers[3], David J. Fermin[1*]

[1] School of Chemistry, University of Bristol, Cantocks Close, BS8 1TS Bristol, United Kingdom.
[2] H. H. Wills Physics Laboratory, University of Bristol, Tyndall Avenue, BS8 1TL Bristol, United Kingdom.
[3] Centre for Renewable Energy Systems Technology (CREST), Wolfson School of Mechanical Electrical and Manufacturing Engineering, Loughborough University, Loughborough, LE11 3TU, United Kingdom

Corresponding author*

David J. Fermin: david.fermin@bristol.ac.uk

**Experimental Methods**

*Preparation of CZTS precursor solution*: CZTS precursor solutions were prepared in a glovebox (Ossila Ltd) at room temperature, with $O_2$ and $H_2O$ levels of less than 15 ppm. For CZTS solution prepared with $ZnCl_2$, the precursor salts were sequentially dissolved in DMF:IPA (75:25 volume ratio) solvent blend in the following order: (1) 2.02 mmol $CuCl_2.2H_2O$ (≥ 99.99%, Sigma Aldrich); (2) 1.22 mmol $SnCl_2$ (≥ 98%, Sigma Aldrich); (3) 1.47 mmol $ZnCl_2$ (≥ 99.99%, Sigma Aldrich) and (4) 23.5 mmol thiourea (TU; 98%, Sigma Aldrich). For CZTS solution prepared with $ZnAc_2$, 2.02 mmol $CuCl_2.2H_2O$ and 23.5 mmol TU were added to vial 1 in DMF:IPA solvent blend, while 1.47 mmol $ZnAc_2.2H_2O$ (≥ 98%, Sigma Aldrich) and 1.22 mmol $SnCl_2$ were added to vial 2 prior to mixing. To maintain a Cu-poor and Zn-rich composition, the molar ratios of Cu/(Sn+Zn), Zn/Sn and TU/metal were 0.75, 1.2 and 5, respectively.

*Fabrication of CZTSSe absorber*: Commercial FTO-coated soda lime glass (SLG) (Ossila Ltd; 2.5 $cm^2$; 500 nm; sheet resistance ~ 10 Ωhm/sq) were cleaned by sonication in deionised water (DI), acetone and DI for 10 minutes each, and then dried using Ar flow. FTO substrates with treated with a UV-$O_3$ plasma (Jelight UVO-Cleaner Model 42 Series) for 20 minutes. For Mo insertion layer, approximately 25 nm Mo was deposited on FTO by direct current (DC) sputtering (AJA International ATC 2200 sputtering system) with 7 sccm Ar and 580 W. CZTS precursor films were prepared in air via spin coating, by dynamically depositing the precursor solution onto FTO at 500 rpm and then increasing to 2000 rpm for 60 s. Immediately following this, the wet film was annealed on a hotplate at 350 ºC for 2 minutes and allowed to cool naturally. This coating-annealing-cooling cycle was repeated 15 times. For devices, the CZTS films were placed into a graphite box, with 28 sccm flow of argon (1 atm), with 600 mg of Se powder and heated to 530 ºC for 20 minutes in a rapid thermal annealing (RTA) furnace (MTI OTF-1200X). To study the grain growth and crystallisation mechanism for $ZnCl_2$- and $ZnAc_2$-based CZTSSe absorbers, selenisation times of 0, 2, 5, 10, 15, 20 and 30 minutes were investigated. Upon selenisation completion, the furnace was allowed to cool naturally to 50 ºC before the removal of the CZTSSe film.

*Device Fabrication*: CZTSSe solar devices on FTO were completed by depositing 50 nm of CdS buffer layer using chemical bath deposition (CBD) from an aqueous bath containing $CdSO_4$, TU and ammonium hydroxide, at 65 ºC. Following this, 50 nm of i-ZnO and 500 nm Al-doped ZnO (AZO) were deposited by radio frequency (RF)-sputtering. Ag (500 nm) top contacts were fabricated by thermal evaporation and solar cells, at an area of 0.25 $cm^2$, were defined by mechanical scribing. Based on the zinc precursor counterion and Mo insertion layer, the absorbers and solar cell devices are referred to as 0-$ZnCl_2$, 25-$ZnCl_2$, 0-$ZnAc_2$ and 25-$ZnAc_2$ in this study.

*Solution Characterisation*: Fourier-transform infrared (FTIR) spectroscopy (PerkinElmer FTIR Spectrometer Spectrum Two) of the precursor solutions was measured between 400 and 4500 $cm^{-1}$ at a resolution of 4 $cm^{-1}$. The background was measured prior and subtracted from the final spectrum, which was measured for a total of 15 scans. Thermogravimetric analysis (TGA) (NETZSCH STA 449 F1 Jupiter) of the individual precursor salts and the dried precursor solutions (~ 10 mg) was measured from 30–800 ºC in an $N_2$ atmosphere, with a heat rate of 5 K $min^{-1}$. To dry the precursor solutions, 500 µL of solution was left on a glass slide to dry over the course of a week to ensure that all solvent had evaporated. The flow rates of pure gas and protective species were 50 mL $min^{-1}$ and 20 mL $min^{-1}$, respectively.

*Film Characterisation*: Top-down and cross-sectional morphology were measured using scanning electron microscopy (SEM) (Jeol IT300 SEM). Surface roughness was measured using confocal laser scanning microscopy (Olympus LEXT OLS5100) at 100 kx. X-ray diffraction (XRD) patterns (2θ = 10-60º, 0.02º, 1 second, 60 rpm) were collected by a Bruker D8 Advance with a PSD LynxEye X-ray powder diffraction system using Cu Kα (λ = 1.54184Å) X-ray source. All spectra were calibrated to FTO (110) peak at 26.7°. Thin film Raman spectra (Renishaw inVia) were acquired using a 488 nm laser excitation source focused into about 1 µm spot using high NA microscope lens. Ultra-high vacuum (UHV) energy-filtered photoemission electron microscopy (EF-PEEM) and X-ray photoelectron spectroscopy (XPS) were performed at the Bristol Ultraquiet NanoESCA Laboratory using a NanoESCA II (ScientaOmicron/FOCUS) EF-PEEM instrument and an Argus (ScientaOmicron) XPS analyser, respectively. For UHV sample preparation, bare CZTSSe absorbers were etched with 0.5 kV $Ar^+$ plasma at 45º for 6 minutes and at a pressure of $1.2\times10^{-5}$ mbar with a total sputter flux of approximately 130 µA min. The energy axis was referenced to the Fermi level of sputtered polycrystalline silver, and all samples did not show any signs of charging during the measurement. The intensity scale was calibrated to parameterised spectra of low-density polyethylene (LDPE). Quantification of metal ratios were performed using Cu $2p_{3/2}$ at 932 eV, Zn $2p_{3/2}$ at 1022 eV and Sn $3d_{5/2}$ at 486 eV. EF-PEEM was performed under UHV conditions (≈ $4\times10^{-11}$ mbar) using monochromatic He I (21.2 eV) as the excitation source. A contrast aperture was inserted into the back focal plane of the microscope for work function maps to improve the lateral resolution, and the energy resolution of the analyser was set to 100 meV at a pass energy of 50 eV.

*Device Characterisation*: Photocurrent density-voltage (J-V) characteristics were measured under AM1.5G conditions using a solar simulator (Wavelabs Sinus-70 light) with an integrated power density of 100 mW $cm^{-2}$. The external quantum efficiency (EQE) spectra were obtained at 0 V bias with 5 nm spectral resolution using a Bentham

PVE300 system, with a monochromator (Bentham TMc300), a dual halogen and single xenon as light sources, and transformer (x500 474 type pre-amp) under half sun conditions.

**Table S1**: FTIR modes between 600 and 2000 $cm^{-1}$ of DMF, TU, and IPA assigned species.

| Species | FTIR ($cm^{-1}$) | Chemical | Assigned mode |
|---|---|---|---|
| O=C–N | 658 | DMF | Bending |
| N–$CH_3$ | 864 | DMF | Symmetric stretching |
| $CH_3$ | 1063 | DMF | Rocking |
| $CH_3N$ | 1091 | DMF | Rocking |
| N–$CH_3$ | 1256 | DMF | Asymmetric stretching |
| C–H | 1385 | DMF | Bending |
| C–N | 1500 | DMF | Stretching |
| C=O | 1660 | DMF | Stretching |
| N–C–N | 486 | TU | Bending |
| N–C–S | 602 | TU | Bending |
| C=S | 740 | TU | Stretching |
| $NH_2$ | 1063 | TU | Rocking |
| C–N/H–N–H | 1400,1458 | TU | Stretching coupled to Bending |
| H–N–H/C–N | 1620 | TU | Stretching coupled to Bending |
| C–C–O | 817 | IPA | Symmetric stretching |
| $CH_3$–C–$CH_3$ | 950 | IPA | Stretching |
| C–C–O | 1129 | IPA | Asymmetric stretching |
| O–H | 1307 | IPA | In-plane bending |
| $CH_3$ | 1467 | IPA | Bending |

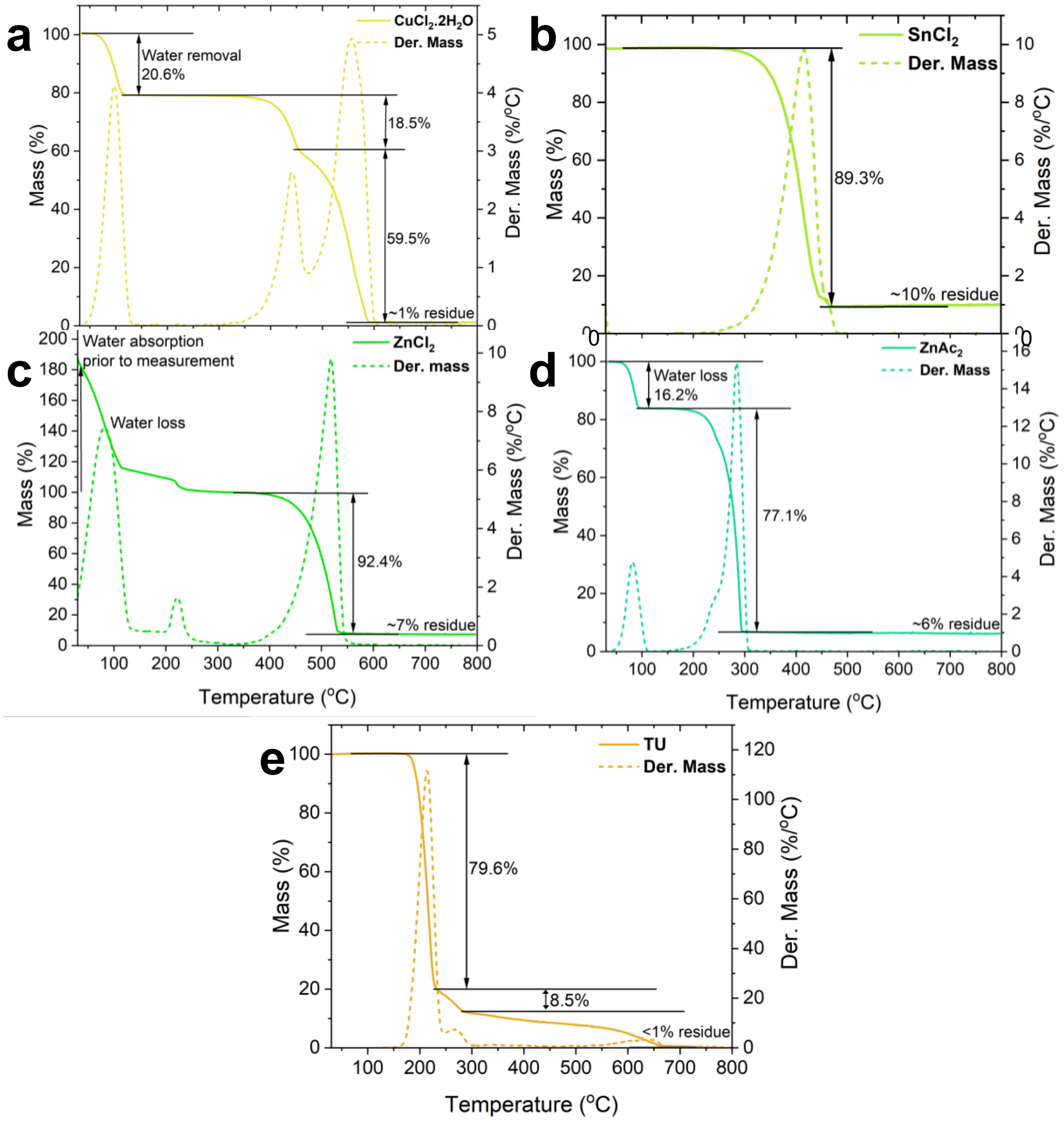


**Figure S1**: TGA analysis of $CuCl_2.2H_2O$ (a), $SnCl_2$ (b), $ZnCl_2$ (c), $ZnAc_2$ (d), and TU (e) between 30 and 800 °C. Der. Mass stands for derived mass, calculated by deriving the mass with respect to temperature.

**Table S2**: TGA decomposition temperatures (°C) of individual precursor salts with % Mass loss and % residue at 800 °C.

| Sample | Decomposition Temperature (°C) | Mass loss (%) | Residue mass (%) |
|---|---|---|---|
| $CuCl_2.2H_2O$ | 442/557 | 18.5/59.5 | < 1 |
| $SnCl_2$ | 417 | 89.3 | 10 |
| $ZnCl_2$ | 519 | 92.4 | 7 |
| $ZnAc_2$ | 284 | 77.1 | 6 |
| TU | 214 | 79.6 | < 1 |

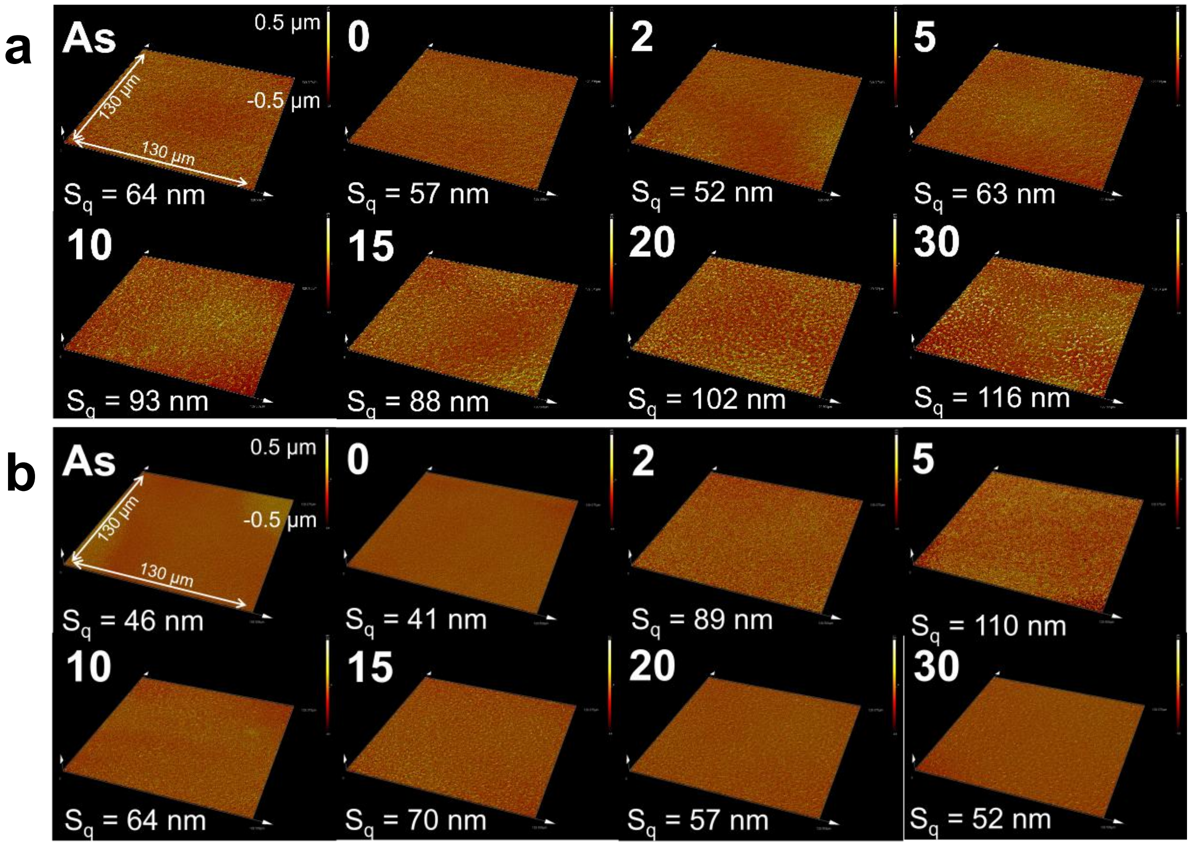


**Figure S2**: Confocal laser scanning microscopy images over 130×130 μm of $ZnCl_2$- (a) and $ZnAc_2$-CZTSSe (b) during selenisation. $S_q$ refers to the root-mean squared roughness.

**Table S3**: Summary of (112) diffraction peak FWHM of CZTSSe extracted from XRD patterns as a function of selenisation time.

| Time (min) | $ZnCl_2$-CZTSSe | $ZnAc_2$-CZTSSe |
|---|---|---|
| As | 3.12 | 4.96 |
| 0 | 2.45 | 2.62 |
| 2 | 0.64 | 0.22 |
| 5 | 0.25 | 0.16 |
| 10 | 0.21 | 0.14 |
| 15 | 0.18 | 0.16 |
| 20 | 0.14 | 0.16 |
| 30 | 0.14 | 0.15 |

**Table S4**: Surface Cu/(Zn+Sn) and Zn/Sn compositions, WF and WF distribution of $ZnCl_2$- and $ZnAc_2$-CZTSSe.

| Sample | Cu/(Zn+Sn) | Zn/Sn | WF (eV) | WF distribution (meV) |
|---|---|---|---|---|
| $ZnCl_2$ | 0.75 | 1.09 | 5.25 | 71 |
| $ZnAc_2$ | 0.69 | 1.53 | 5.28 | 85 |

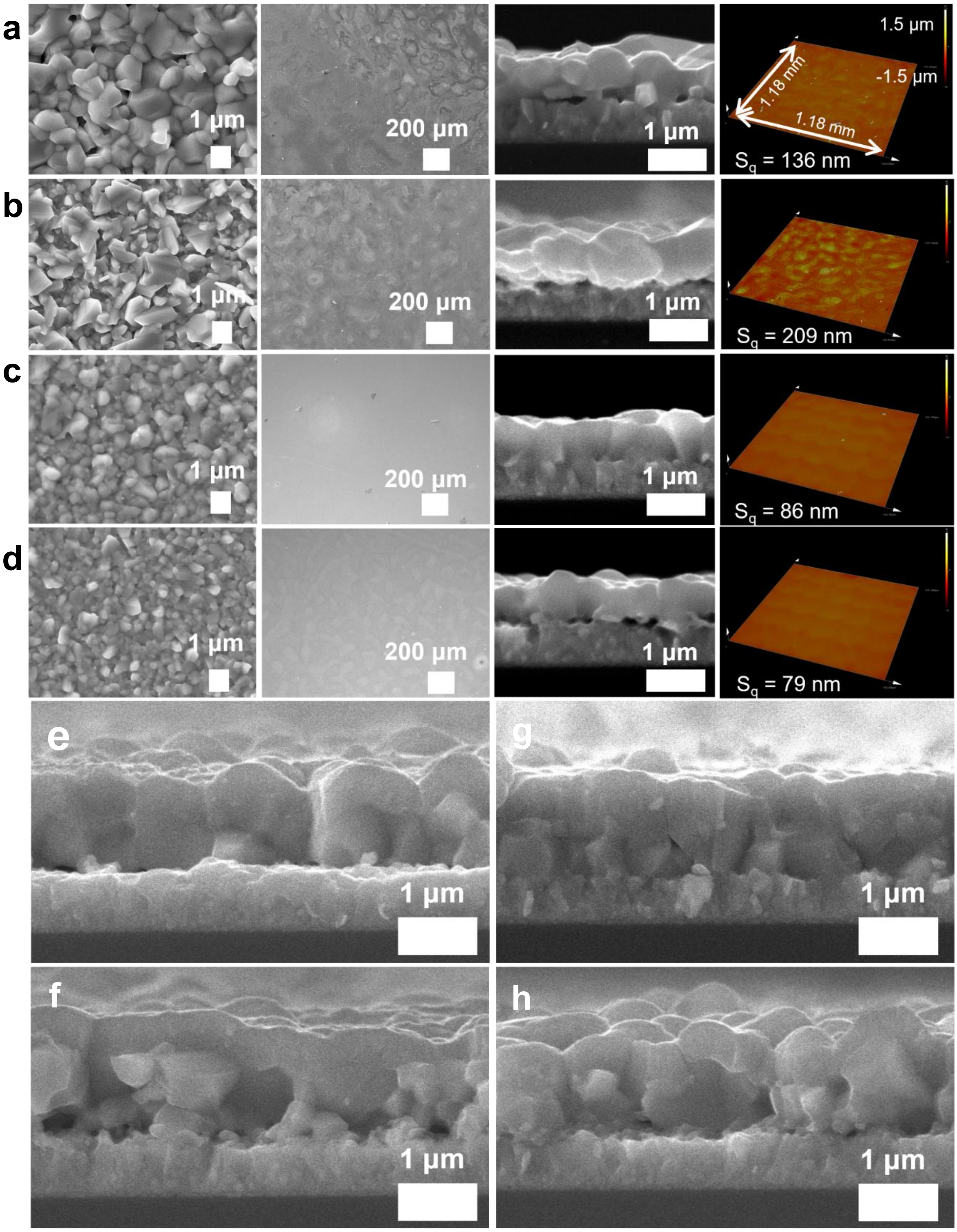


**Figure S3**: Top-down SEM, at 10 kx (left) and 75 x (left middle), cross-sectional SEM (right middle) and surface roughness analysis over 1.18×1.18 mm (right) of $ZnCl_2$-CZTSSe without (0-$ZnCl_2$) (a) and with 25 nm Mo layer (25-$ZnCl_2$) (b) and $ZnAc_2$-CZTSSe without (0-$ZnAc_2$) (c) and with 25 nm Mo layer (25-$ZnAc_2$) (d) after 20 minutes of annealing. Cross-sectional SEM images of 0-$ZnCl_2$ (e), 25-$ZnCl_2$ (f), 0-$ZnAc_2$ (g) and 25-$ZnAc_2$ (h) devices, with SLG/FTO/Mo (0 or 25 nm)/CZTSSe/CdS/i-ZnO/Al:ZnO/Ag configuration.

**Table S5**: Photovoltaic parameters of champion cell (0.25 $cm^2$) for $ZnCl_2$- and $ZnAc_2$-based CZTSSe devices under front illumination.

| Condition | PCE (%) | $J_{sc}$ (mA $cm^{-2}$) | $V_{OC}$ (V) | FF (%) | $R_s$ (Ω $cm^2$) | $R_{sh}$ (Ω $cm^2$) | n |
|---|---|---|---|---|---|---|---|
| 0-$ZnCl_2$ | 3.27 | 21.5 | 0.338 | 45.0 | 1.9 | 62.8 | 2.09 |
| 25-$ZnCl_2$ | 2.62 | 21.3 | 0.357 | 34.5 | 4.6 | 39.8 | 2.76 |
| 0-$ZnAc_2$ | 4.49 | 26.8 | 0.376 | 44.7 | 3.1 | 83.8 | 2.15 |
| 25-$ZnAc_2$ | 6.02 | 26.1 | 0.403 | 57.3 | 1.9 | 331 | 1.63 |

**Table S6**: Mean photovoltaic parameters of (0.25 $cm^2$) for $ZnCl_2$- and $ZnAc_2$-based CZTSSe devices under front illumination.

| Condition | PCE (%) | $J_{sc}$ (mA $cm^{-2}$) | $V_{OC}$ (V) | FF (%) | $R_s$ (Ω $cm^2$) | $R_{sh}$ (Ω $cm^2$) |
|---|---|---|---|---|---|---|
| 0-$ZnCl_2$ | 1.98 ± 0.87 | 19.3 ± 2.4 | 0.283 ± 0.070 | 33.9 ± 6.4 | 2.93 ± 0.60 | 47.0 ± 21.0 |
| 25-$ZnCl_2$ | 1.30 ± 0.75 | 16.8 ± 2.8 | 0.228 ± 0.081 | 29.1 ± 3.3 | 3.55 ± 0.80 | 27.6 ± 9.7 |
| 0-$ZnAc_2$ | 2.62 ± 1.00 | 21.5 ± 2.7 | 0.337 ± 0.029 | 34.9 ± 5.8 | 3.04 ± 0.26 | 40.6 ± 20.0 |
| 25-$ZnAc_2$ | 5.19 ± 0.57 | 26.3 ± 2.2 | 0.395 ± 0.011 | 49.9 ± 3.9 | 2.97 ± 0.70 | 212.1 ± 84.1 |

**Table S7**: Photovoltaic parameters of champion cell (0.25 $cm^2$) for $ZnCl_2$- and $ZnAc_2$-based CZTSSe devices under rear illumination.

| Condition | PCE (%) | $J_{sc}$ (mA $cm^{-2}$) | $V_{OC}$ (V) | FF (%) |
|---|---|---|---|---|
| 0-$ZnCl_2$ | 0.01 | 2.0 | 0.019 | 23.0 |
| 25-$ZnCl_2$ | 0.02 | 2.6 | 0.026 | 23.9 |
| 0-$ZnAc_2$ | 0.61 | 8.3 | 0.240 | 30.7 |
| 25-$ZnAc_2$ | 0.59 | 4.6 | 0.311 | 41.6 |

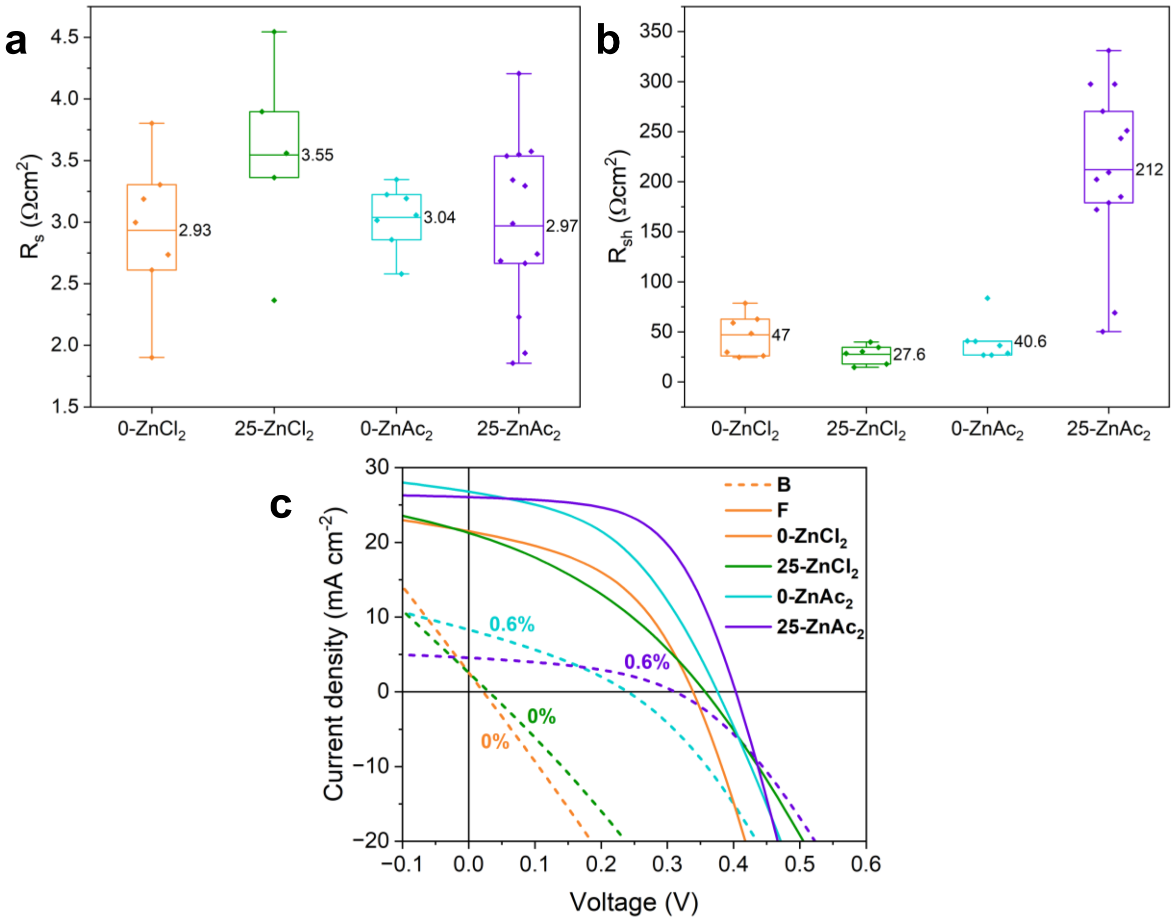


**Figure S4**: Series (a) and shunt (b) resistances of 0-$ZnCl_2$ (orange), 25-$ZnCl_2$ (green), 0-$ZnAc_2$ (aqua) and 25-$ZnAc_2$ (purple) CZTSSe devices. J–V analysis of back (B – dashed) and front (F – solid) illumination CZTSSe devices (c).
